# Tuberculose multifocale simulant un cancer du côlon multi-métastatique chez un Noir africain immunocompétent: à propos d´un cas

**DOI:** 10.11604/pamj.2021.39.167.26879

**Published:** 2021-07-02

**Authors:** Aboudou Raïmi Kpossou, Sonia Adjadohoun, Kadiatou Diallo, Salim Badarou, Gabriel Ngamo, Comlan N’déhougbèa Martin Sokpon, Rodolph Koffi Vignon, Romulus Takin, Patricia Yekpè, Jean Sehonou, Olivier Biaou

**Affiliations:** 1 Clinique Universitaire d’Imagerie Méficale, Centre National Hospitalier Universitaire Hubert Koutoukou Maga (CNHU-HKM), Cotonou, Bénin,; 2 Service de Médecine Interne, Hôpital Donka, Conakry, Guinée,; 3 Laboratoire d’Anatomie Pathologique, Centre de Cancérologie de Cotonou, Cotonou, Bénin

**Keywords:** Tuberculose multifocale, diagnostic, Bénin, à propos d’un cas, Multifocal tuberculosis, diagnosis, Benin, case report

## Abstract

La tuberculose multifocale est rare chez le sujet immunocompétent. Caractérisée par une atteinte d´au moins deux sites extra-pulmonaires, associée ou non à une atteinte pulmonaire, elle est souvent de diagnostic difficile. Nous rapportons un cas de tuberculose multifocale chez un sujet noir africain non immunodéprimé au Centre National Hospitalier et Universitaire Hubert Koutoukou Maga (CNHU-HKM) de Cotonou au Bénin. Il s´agissait d´un jeune homme de 23 ans, sans antécédent particulier, admis pour une douleur abdominale diffuse, associée à une altération de l´état général. L´examen clinique objectivait un état de dénutrition sévère et une ascite de moyenne abondance. L´imagerie à travers les différentes modalités (radiographie du thorax, échographie et tomodensitométrie (TDM)) a objectivé de multiples lésions pulmonaires, hépatiques, pancréatiques, osseuses, ganglionnaires et coliques ayant d´abord fait suspecter un processus tumoral multi-métastatique. Une coloscopie avait ensuite montré une lésion bourgeonnante du caecum, dont les biopsies ont permis de mettre en évidence des Bacilles de Koch par GeneXpert. L´examen anatomo-pathologique des biopsies coliques et le GeneXpert des crachats avaient permis de confirmer la tuberculose multifocale. Mis sous traitement antituberculeux et soutien nutritionnel, l´évolution était marquée par le décès du patient. La tuberculose multifocale demeure une maladie grave, de diagnostic difficile, source d´errance diagnostique en milieu tropical d´autant plus si elle survient sur un terrain immunocompétent.

## Abstract (en)

Multifocal tuberculosis is rare in immunocompetent subjects. It is characterized by the involvement of at least two extra-pulmonary sites, associated or not with lung disease. It is often difficult to diagnose. We here report a case of multifocal tuberculosis in a non-immunocompromised black African subject at the Hubert Koutoukou Maga National Hospital and University Center (CNHU-HKM) in Cotonou, Benin. The study involved a 23-year-old man, with no particular previous history, admitted with diffuse abdominal pain associated with alteration of general state. Clinical examination showed severe malnutrition and medium-volume ascites. Imaging tests (chest X-ray, ultrasound and computed tomography (CT) scan) showed multiple lung, liver, pancreatic, bone, lymph nodes and colic lesions suggesting multimetastatic tumor. Colonoscopy then showed budding lesion of the cecum. GeneXpert test showed Koch´s bacilli. The anatomo-pathological examination of colic biopsies and GeneXpert sputum test confirmed multifocal tuberculosis. The patient received antituberculosis treatment and nutritional support. However he died. Multifocal tuberculosis is a serious disease that is difficult to diagnose. Then it is frequently mis-diagnosed in tropical areas, especially when it occurs in immunocompetent patients.

## Introduction

La tuberculose (TB) est une maladie infectieuse due au bacille *Mycobacterium tuberculosis* (MT). Elle atteint préférentiellement les poumons, mais les localisations extra-pulmonaires sont fréquentes [1,2]. La TB est une des principales causes de morbidité et de mortalité dans les pays en voie de développement, comme le Bénin [1]. La tuberculose est dite multifocale lorsqu´il y a l´atteinte d´au moins deux sites extra-pulmonaires non contigus associée ou non à une atteinte pulmonaire [3]. Elle représente 9 à 10% des tuberculoses extra-pulmonaires [3]. C´est une forme grave et rare puisque la mortalité atteint 16 à 25% [3]. Son aspect multiple est souvent trompeur et peut égarer le diagnostic [4,5]. Elle est surtout l´apanage des sujets immunodéprimés, principalement des personnes infectées au VIH. Toutefois, elle peut survenir chez un sujet immunocompétent et être source d´errance diagnostique en milieu tropical. C´est le cas du patient, objet de cet article dont la présentation clinique et radiologique de tuberculose multifocale simulait un cancer du côlon multi-métastatique.

## Patient et observation

**Présentation du patient**: patient de 23 ans, béninois, étudiant en faculté de langue, habitant à Abomey-Calavi, sans antécédent médical ou chirurgical particulier. Orphelin de mère et abandonné par son père, il est resté en orphelinat pendant une dizaine d´années. Il a été admis à la clinique universitaire d´Hépato-gastroentérologie pour une altération de l´état général et des douleurs abdominales. L´interrogatoire a noté un début remontant à environ 15 ans par des douleurs abdominales péri-ombilicales, d´intensité modérée n´irradiant pas, associées à des vomissements alimentaires postprandiaux et une diarrhée intermittente non glaireuse et non sanglante. Une endoscopie digestive haute avait été réalisée et révélait une gastrite à *Helicobacter pylori* pour laquelle un traitement a été fait. L´évolution a d´abord été marquée par une régression des signes puis par une reprise de la douleur abdominale avec un œdème des membres pelviens, une augmentation du volume abdominal, une bouffissure du visage, une constipation, une fièvre, un amaigrissement important, une asthénie et une anorexie. Ce qui motiva une nouvelle consultation d´abord dans un centre privé, puis sa référence au CNHU-HKM. Après un suivi au service d´hématologie pour une suspicion de lymphome non Hodgkinien, il a été transféré dans la clinique universitaire d´Hépato-gastroentérologie pour la poursuite de la prise en charge. Il n´avait pas d´ictère, d´hématémèse, de méléna, ni de rectorragie.

**Résultats cliniques**: à l´examen on trouvait un état général altéré avec l´indice de Performance Status de l´OMS à 3, les muqueuses normo-colorées, un œdème des membres pelviens bilatéraux ne prenant pas le godet jusqu´au 1/3 inférieur, mou, indolore. Le poids à 43 kg pour une taille de 1m77, soit un indice de masse corporelle (IMC) à 13,7 Kg/m^2^ la température à 37,6°C, la fréquence respiratoire à 15 cycles/mn, la tension artérielle à 97/70 mmHg, le pouls à 100 pulsations/mn. A l´examen hépato-digestif, à la cavité buccale on trouve une muqueuse normale, une langue propre, une dentition complète et une bonne haleine. On ne trouvait pas de voussure abdominale ni de scarification ni de signe cutané d´insuffisance hépatocellulaire. On notait un abdomen augmenté de volume, l´ombilic plissé, une hépatomégalie douloureuse, surface régulière, consistance ferme, une matité des flancs avec tympanisme péri-ombilical. L´examen proctologique est normal sans masse palpable au niveau des parois ano-rectales. L´examen cardio-vasculaire est normal ainsi que les examens pleuro-pulmonaires et uro-génitaux. L´examen ostéo-articulaire trouvait un syndrome rachidien dorso-lombaire (une cyphose lombaire haute et dorsale basse, une spinalgie palpatoire lombaire basse, une extension lombaire douloureuse). Il n´y avait pas de ganglion de Troisier ni d´autre adénopathie périphérique, ni de splénomégalie à l´examen spléno-ganglionnaire. L´examen neurologique notait une conscience normale, des pupilles isochores normo-réactives, une motricité et une sensibilité normales, des réflexes ostéo-tendineux et cutanéo-plantaires bilatéraux, symétriques normaux.

**Evaluation diagnostique**: sur le plan biologique, l´hémogramme affichait une anémie (Hémoglobine=6,6 g/dL Hématocrite=21,6%) microcytaire (volume globulaire moyen=69 fl) arégénérative (Réticulocyte=33,2 Giga/lite (G/L)) avec hyperleucocytose à 11,2 G/L à prédominance neutrophile 9,184 G/L, plaquette normale à 353 G/L; taux de prothrombine bas à 45,7%, ASAT=55 UI/L, ALAT= 52 UI/L, Bilirubine totale à 3 mg/L, hypo albuminémie 31 g/L, hypergammaglobulinémie polyclonale, LDH =194 UI/L; sérologie VIH (virus de l´immunodéficience humaine) négative, Antigène HBs et anticorps anti-VHC (virus de l´hépatite C) négatifs; alphafoetoprotéine à 2,18 ng/ml, CA19-9 à 8 UI/ml, ACE à 1,66 ng/ml; amylasémie 156 UI/L, lipasémie 52 UI/L; C-Réactive protéine à 375,6 mg/L. La réaction en chaîne par polymérase (PCR) de *Mycobacterium tuberculosis* des crachats était positive. L´électrophorèse des protides notait: une protidémie normale à 75 g/L, une hypoalbuminémie à 41,4 g/L, une hypergammaglobulinémie polyclonale. L´examen du liquide d´ascite notait un liquide pauvre en protéine à 17 g/L avec à l´ examen cytobactériologique (ECB) des leucocytes à 54/mm^3^ et une culture négative.

**Figure 1:**
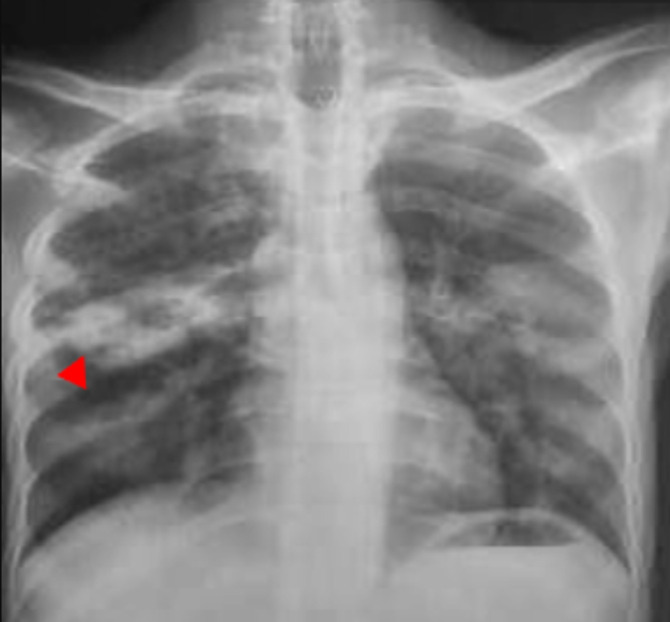
radiographie pulmonaire face montrant une image cavitaire avec une paroi opaque para-hilaire droite (tête de flèche), avant le traitement

**Figure 2:**
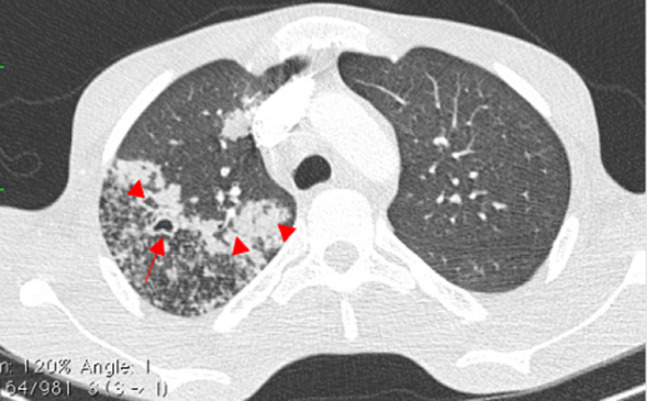
coupe axiale TDM thoracique en fenêtre pulmonaire mettant en évidence des foyers de condensations alvéolaires (têtes de flèche) avec une lésion excavée apicale droite (flèche)

**Figure 3:**
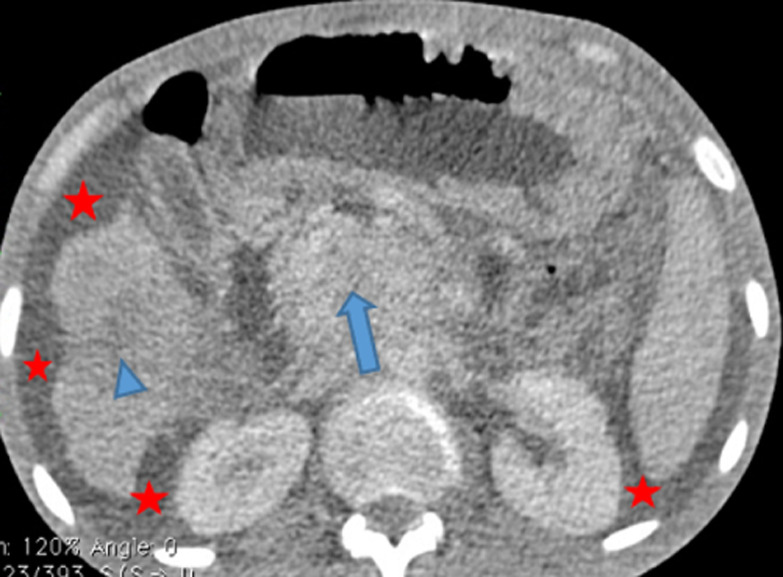
coupe axiale TDM abdomino-pelvienne avec injection de produit de contraste iodé: hépatomégalie multi nodulaire (tête de flèche bleu) masse du pancréas (flèche bleu), avec une ascite de grande abondance (étoiles rouges)

**Figure 4:**
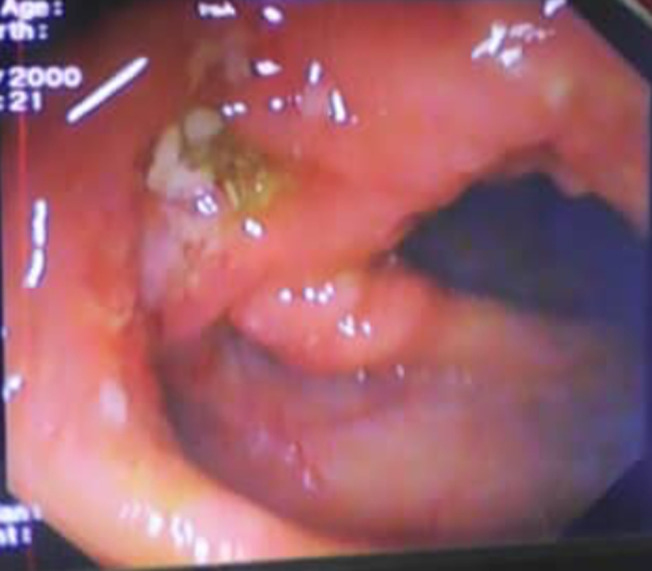
coloscopie totale avec masse ulcéro-bourgeonnante du colon droit

Sur le plan radiologique, la radiographie pulmonaire de face (Figure 1), montrait une image cavitaire dans la région para-hilaire du poumon droit. L´échographie abdominale notait une légère hépatomégalie, de contours bosselés, hétérogène multi-nodulaire avec la lésion la plus volumineuse mesurant 28 mm dans le segment III, une splénomégalie homogène; un épaississement pariétal de quelques anses digestives notamment en fosse iliaque droite, des adénopathies coelio-mésentériques et un épanchement intra-péritonéal de moyenne abondance. Le scanner thoraco-abdomino-pelvien montrait des lésions multi-viscérales notamment des adénomégalies médiastinales et abdominales, pleurales, pulmonaires (Figure 2), hépatiques, pancréatiques (Figure 3), osseuses, avec de l´ascite faisant évoquer des localisations secondaires d´un processus tumoral de primitif indéterminé. Il existait par ailleurs une prise de contraste en accordéon des parois coliques faisant évoquer une colite associée. À l´endoscopie haute on ne retrouvait pas de lésion, mais la coloscopie montrait une tumeur ulcéro-bourgeonnante (Figure 4) dont la PCR (GeneXpert) du prélèvement biopsique montre la présence de BK. L´analyse anatomo-pathologique de ce prélèvement concluait à une colite granulomateuse, son aspect sans être spécifique peut être compatible avec une mycobactériose et il n´y avait pas de signe de malignité. La recherche de BK dans les crachats par PCR était positive. Le diagnostic de tuberculose multifocale était retenu.

**Intervention thérapeutique**: le patient était gardé en hospitalisation et un traitement antituberculeux à base de la combinaison fixe Ethambutol 275 mg, Rifampicine 150 mg, Isoniazide 75 mg, Pyrazinamide 400 mg (ERHZ) à la dose de 3 comprimés per os le matin à jeun a été institué. La prise du traitement était supervisée par un infirmier. Une assistance nutritionnelle par des compléments alimentaires oraux et un suivi diététique ont été assurés.

**Figure 5:**
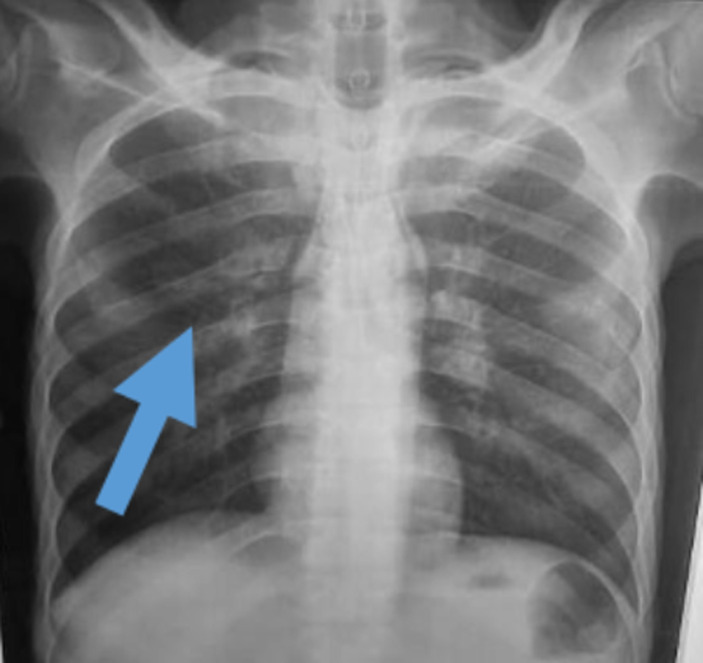
radiographie pulmonaire au bout de 6 semaines de traitement antituberculeux régression de la lésion cavitaire

**Suivi et résultats des interventions thérapeutiques**: l´évolution a été initialement très lentement favorable. Le contrôle de l´examen des crachats à 1 mois était négatif, et la radiographie du thorax (Figure 5) après 6 semaines montrait une nette régression de l´image cavitaire. Au bout de 2 mois d´hospitalisation, le patient a présenté brutalement une détresse respiratoire à la suite d´une inhalation alimentaire broncho-pulmonaire et en est décédé, malgré les gestes de réanimation.

**Point de vue du patient**: le patient était satisfait du traitement qui lui a été prescrit et l´observance thérapeutique était bonne.

**Consentement éclairé**: le consentement éclairé oral du patient a été obtenu.

## Discussion

La particularité de ce cas clinique réside en plusieurs points. La TB multifocale est une affection rare; elle survient le plus souvent sur un terrain immunodéprimé, le sujet dans ce cas clinique était immunocompétent. Peu connue, la TB multifocale peut être source d´errance diagnostique en Afrique tropicale, et simulée une pathologie tumorale multi-métastatique comme dans le cas clinique que nous avons rapporté. L´atteinte colique est une localisation rare qui renforce la particularité de ce cas clinique. La dénutrition sévère était remarquable chez ce patient et il était difficile de préciser si elle était un facteur favorisant ou une conséquence de la TB multifocale chez ce sujet en précarité sociale. La tuberculose multifocale représente 9 à 10% des atteintes extra-pulmonaires [3]. Elle siège par ordre de fréquence décroissant, au niveau ganglionnaire, génito-urinaire, ostéo-articulaire et neuro-méningé [6,7]. La localisation abdominale est relativement fréquente et représente 5 à 10% de l´ensemble des localisations [7]. La tuberculose multifocale est rare chez un sujet immunocompétent. Il a été démontré que le risque de développer des atteintes extra-pulmonaires est proportionnel au degré du déficit immunitaire [3]. Chez notre patient dont la sérologie VIH est négative, l´atteinte multifocale pourrait s´expliquer par le retard au diagnostic chez un sujet relativement immunodéprimé par la dénutrition favorisée par les difficultés sociales et ayant vécu longtemps en communauté. Les localisations ont alors été multiples: pulmonaire, ganglionnaire, colique, pancréatique, hépatique, et osseuse. Plusieurs hypothèses ont été avancées pour expliquer les formes graves de tuberculose chez les immunocompétents. Certains auteurs ont pu établir une liaison entre la tuberculose diffuse et l´intensité de la transmission dans la collectivité [8]. Un syndrome de la susceptibilité mendélienne aux infections mycobactériennes est décrit et neuf gènes ont été identifiés comme responsables [9]. Le retard diagnostique s´explique ici par le tableau clinique polymorphe et en l´absence initiale de signes évocateurs de TB notamment la toux chronique, la transpiration nocturne et la fièvre. Par ailleurs, devant l´altération de l´état général, le syndrome douloureux abdominal, les troubles du transit, le syndrome rachidien, et le scanner thoraco-abdomino-pelvien qui montrait des lésions décrites comme tumorales secondaires au niveau du poumon, du foie, du pancréas, ganglionnaire et osseux; un cancer digestif (colique) métastatique ou une hémopathie maligne (lymphome) étaient d´abord évoqués. Une coloscopie totale alors réalisée avait découvert une masse ulcéro-bourgeonnante au niveau du colon droit dont l´examen anatomopathologique et la PCR (GeneXpert) des prélèvements biopsiques ont permis d´écarter la malignité et de retenir le diagnostic de TB multifocale. Ce diagnostic a ensuite été corroboré par la PCR des crachats et la radiographie pulmonaire.

L´imagerie médicale dans ce cas clinique, notamment le scanner thoraco-abdomino-pelvien a permis de faire le bilan lésionnel et a orienté vers une atteinte systémique, sans pour autant définir une entité particulière. Les lésions excavées pulmonaires lobaires supérieures à la radiographie pulmonaire et les adénopathies médiastinales étaient très évocatrices sémiologiquement d´une lésion granulomateuse de tuberculose ou de sarcoïdose. Ce cas est similaire à celui d´une TB multifocale inhabituelle rapportée à Bobo Dioulasso par Bicaba *et al*. [10]. La tuberculose a été suspectée au scanner thoraco-abdomino-pelvien par une association lésionnelle qui comprenait une volumineuse collection liquidienne abdomino-pelvienne sous péritonéale impure, encapsulée, à paroi fine et régulière, évaluée à 08 litres associée à des adénopathies médiastinales nécrosées en amas non compressives, sans adénopathies ni de masse tumorale abdomino-pelvienne, ainsi que de lésion parenchymateuse pulmonaire. Le scanner peut ainsi avoir un rôle de boussole dans la prise en charge et permet d´avoir un large spectre de diagnostics différentiels. L´apport de l´endoscopie est essentiel, elle permet la détection des lésions, même les plus superficielles [11]. Son intérêt majeur est qu´elle permet la réalisation de biopsies avec études histologique et bactériologique (culture) et évite alors la morbidité et la mortalité liées à une laparotomie exploratrice [6]. La coloscopie peut montrer soit des érosions muqueuses, des ulcérations avec des zones nécrotico-hémorragiques, soit des zones de rétrécissement ou de sténose infranchissable avec des bourgeons autour. Dans notre cas clinique elle a permis de trouver une lésion ulcéro-bourgeonnante au niveau du colon droit. L´examen anatomo-pathologique quant à lui a aussi joué un rôle très important dans le diagnostic dans le sens où il a permis de caractériser la nature de la lésion colique droite découverte grâce à la coloscopie. Il a identifié un granulome épithélioïde qui est un fort argument en faveur du diagnostic de TB [12]. Cependant, il n´y avait pas la classique nécrose caséeuse. Dans la littérature la nécrose centrale caséeuse n´est pas constante [13]. Lorsqu´elle est présente elle est très évocatrice mais non spécifique. Les examens biologiques, une fois la suspicion de tuberculose établie ont permis de conforter le diagnostic. Il s´agit de la coloration de Ziehl-Neelsen à la recherche de bacilles acido-alcoolo-résistants (BAAR) dans les crachats revenue positive. C´est une coloration peu performante et sa négativité n´élimine pas le diagnostic. Il s´agit également de la GeneXpert, méthode d´amplification génétique qui permet d´identifier spécifiquement les bacilles de Koch (BK) sans que la culture bactérienne ne soit nécessaire, notant également la présence de BK [13].

L´atteinte colique qui a permis d´aboutir au diagnostic est une localisation rare. Elle s´est faite probablement par voie hématogène à partir de l´atteinte pulmonaire. En effet, au cours de la tuberculose abdominale tous les organes peuvent être atteints, et les localisations les plus habituelles sont l´intestin grêle (44%), le caecum (35%) et l´iléo-caecum (16%) [14]. L´atteinte isolée du côlon est rare. Elle est estimée à 10,8% et dominée par l´atteinte du côlon droit comme retrouvé dans notre cas clinique [15,16]. La tuberculose colique se manifeste souvent par des douleurs abdominales (90%), un amaigrissement (65-75%), une fièvre (35-50%) ou une diarrhée (25-50%). Elle peut être diagnostiquée au stade de complication: occlusion intestinale (15 à 60%), perforation (15%) ou fistules digestives (25%); les rectorragies massives sont exceptionnelles [16]. Dans notre cas clinique aucune de ces complications n´a été objectivée. La localisation iléo-colique pose surtout le problème du diagnostic différentiel avec la maladie de Crohn, qui est tranché par l´examen anatomo-pathologique [16]. Les autres localisations abdominales plus courantes dans cette forme diffuse de tuberculose concernent les ganglions, le péritoine, le foie et la rate [17]. La localisation ganglionnaire est la plus fréquente dans la littérature contribuant facilement au diagnostic par son accessibilité à l´examen anatomo-pathologique [3]. L´atteinte du pancréas est moins fréquente. Dans la littérature des formes primitives existent et sont isolées dans 25% des cas [18]. Par ailleurs, la localisation ostéo-articulaire, notamment vertébrale est plus fréquente [19]. Elle représente 2 à 5% des tuberculoses et 11 à 15% des tuberculoses extra-pulmonaires [20].

Le patient a alors reçu un traitement antituberculeux fait de l´association ERHZ (Ethambutol Rifampicine Isoniazide Pyrazinamide). Au bout de 2 mois de traitement les résultats étaient satisfaisants: sur le plan clinique avec une amélioration de l´état général, la reprise correcte de l´alimentation, et sur le plan paraclinique avec la PCR de contrôle des crachats négatif à un mois, la radiographie pulmonaire de contrôle qui montrait une nette disparition de la caverne tuberculeuse (Figure 5). Toutefois, le patient décéda brutalement d´une inhalation broncho-pulmonaire. Ce cas clinique nous montre que la TB multifocale malgré sa rareté reste une pathologie grave en milieu tropical; il est important d´y penser même chez le patient immunocompétent et de réaliser un bilan paraclinique bien hiérarchisé, incluant le scanner thoraco-abdomino-pelvien pour le bilan d´extension des lésions. La biopsie des lésions avec examen anatomo-pathologique et la PCR (GeneXpert) sont les examens clés pour confirmer le diagnostic de la TB.

## Conclusion

La tuberculose multifocale est une forme grave qui touche le plus souvent les sujets immunodéprimés. Néanmoins elle peut toucher des sujets immunocompétents comme le cas objet de cet article. La dénutrition favorise l´atteinte multifocale. Le diagnostic est souvent difficile et retardé. Le scanner permet de réaliser un bilan lésionnel précis. La coloscopie avec la PCR du MT et l´examen anatomo-pathologique des biopsies coliques ont été déterminants dans le diagnostic. Il s´avère nécessaire de rechercher le MT dans toutes les situations d´atteinte multi viscérale pouvant faire suspecter une pathologie maligne ou une maladie du système.
